# Honokiol, a Polyphenol Natural Compound, Attenuates Cisplatin-Induced Acute Cytotoxicity in Renal Epithelial Cells Through Cellular Oxidative Stress and Cytoskeleton Modulations

**DOI:** 10.3389/fphar.2018.00357

**Published:** 2018-04-24

**Authors:** Tse-En J. Wang, Hung-Ting Liu, Yu-Hua Lai, Tong-Rong Jan, Naohiro Nomura, Hui-Wen Chang, Chi-Chung Chou, Ya-Jane Lee, Pei-Shiue J. Tsai

**Affiliations:** ^1^Department of Veterinary Medicine, School of Veterinary Medicine, National Taiwan University, Taipei, Taiwan; ^2^Graduate Institute of Veterinary Medicine, School of Veterinary Medicine, National Taiwan University, Taipei, Taiwan; ^3^Department of Nephrology, Graduate School of Medical and Dental Sciences, Tokyo Medical and Dental University, Tokyo, Japan; ^4^Graduate Institute of Molecular and Comparative Pathobiology, School of Veterinary Medicine, National Taiwan University, Taipei, Taiwan; ^5^Department of Veterinary Medicine, College of Veterinary Medicine, National Chung Hsing University, Taichung, Taiwan; ^6^Graduate Institute of Veterinary Clinical Science, School of Veterinary Medicine, National Taiwan University, Taipei, Taiwan; ^7^Research Center for Developmental Biology and Regenerative Medicine, National Taiwan University, Taipei, Taiwan

**Keywords:** honokiol, cisplatin, kidney, cytoskeleton, oxidative stress

## Abstract

Cisplatin is a potent anti-cancer drug that has been widely used in the treatment of various cancers; however, cisplatin administration results in severe nephrotoxicity and impedes its clinical applications. In this study, we showed that honokiol, a polyphenol constituent extracted from *Magnolia officinalis* exhibited a short-term protective effect against cisplatin-induced damages in renal epithelial cells *in vitro*. The protective effects of honokiol were resulted from the combination of (1) reduced cellular oxidative stress ranging from 53 to 32% reduction during a 24-h incubation, (2) the maintenance of cellular antioxidant capacity and (3) the stabilization of cytoskeletal structure of the kidney epithelial cells. By promoting the polymerization of actin (1.6-fold increase) and tubulin (1.8-fold increase) cytoskeleton, honokiol not only maintained epithelial cell morphology, but also stabilized cellular localizations of tight junction protein Occludin and adhesion junction protein E-Cadherin. With stabilized junction protein complexes and structural polymerized cytoskeleton network, honokiol preserved epithelial cell polarity and morphology and thus reduced cisplatin-induced cell disruption and damages. Our data demonstrated for the first time that honokiol could counteract with cisplatin-induced damages in renal epithelial cells *in vitro*, future *in vivo* studies would further validate the potential clinical application of honokiol in cisplatin-based cancer treatments with reduced nephrotoxicity.

## Introduction

The kidney is an important organ responsible for waste elimination, electrolyte balance and erythropoietin production (Brown et al., 2012). Clinical symptoms for renal dysfunction include proteinuria, hypokalemia, metabolic acidosis and hyperphosphatemia (Chakrabarti et al., 2012). Both acute kidney injury (AKI) and chronic kidney disease (CKD) are characterized with different levels of functional or structural abnormalities of renal tissues. Kidney consists of various tubule structure formed by a single layer of epithelial cells that their apical membranes face toward the lumen where urine/water, endogenous waste and hormones are deposited to or secreted into (Brown et al., 2012), and their lateral membranes of adjacent renal epithelial cells are connected by junction protein complexes (Giepmans and van Ijzendoorn, 2009). To maintain this spatial segregation and orientation, epithelial cells must establish their cellular polarity. The generation of cell polarity is mediated by cell–cell adhesion and cell attachment to the extracellular matrix (Yeaman et al., 1999; Martin-Belmonte et al., 2008). Furthermore, the synthesis of signaling adhesion protein complexes at the sites of cell junctions, the organization of cytoskeleton and the constitutive sorting and transportation of proteins and molecules toward different membrane domains all contribute to the functional specialization of these asymmetry membrane regions and the maintenance of cell polarity (Giepmans and van Ijzendoorn, 2009). Alterations of kidney epithelial cell polarity have been reported in various pathological processes (Fish and Molitoris, 1994) and loss of cell polarity in kidney tubules upon the occurrence of AKI and CKD results in disruption of basic kidney tubular structure and defects of vital physiological functions (Thadhani et al., 1996).

Cisplatin has been used as an anti-cancer drug since 1970s (Dasari and Tchounwou, 2014). By forming inter- and intra-strand DNA adducts, cisplatin leads to apoptosis and necrosis of highly proliferating tumor cells (Chaney et al., 2005; Garcia Sar et al., 2012). Although cisplatin is one of the most effective platinum-containing therapeutic agents in treating various cancers (Desoize and Madoulet, 2002), its renal toxicity often impedes its clinical applications. The mechanism of cisplatin-induced cytotoxicity and organ failure is not only due to DNA damages of the cells, oxidative and nitrosative stresses induced by cisplatin have also been suggested (Santos et al., 2007; Dasari and Tchounwou, 2014). Cisplatin has been shown to provoke mitochondrial dysfunction that interfered the electron transport system and thus enhanced the generation of reactive oxygen species (ROS), nitrogen species (RNS) and led to mitochondrial dysfunction (Choi et al., 2015). Oxidative stress (OS) resulted from excessive amount of ROS and RNS generated by cisplatin is thought to participate in the pathogenesis of glomerular and tubular injuries in various renal diseases (Ozkok and Edelstein, 2014; Sureshbabu et al., 2015).

Defense mechanisms that protect cells from ROS-induced cell damage are crucial in both human and animal health. Besides traditional antioxidants (e.g., vitamin C or E), a number of exogenous antioxidants have also been used to against aging and neurodegeneration-related diseases (Youdim and Joseph, 2001; Agarwal et al., 2014). Among those of non-enzymatic antioxidants, honokiol (HNK), a small-molecule polyphenol constituent extracted from the bark of *Magnolia officinalis* exhibits several bioactivities including anti-allergy (Munroe et al., 2010), anti-anxiety (Guillermo et al., 2012; Sulakhiya et al., 2015), anti-cancer (Bai et al., 2003; Dasari and Tchounwou, 2014; Cheng et al., 2016), anti-depression (Jangra et al., 2015), and neuroprotection (Matsuda et al., 2001; Shen et al., 2010). Recent *in vitro* and *in vivo* studies showed that HNK exhibited anti-inflammation and anti-oxidant properties (Kim and Cho, 2008; Chao et al., 2010; Munroe et al., 2010; Li et al., 2011). Moreover, HNK is proved to be a multifunctional anti-oxidative molecule via its ability to reduce ROS production (Shen et al., 2010). On the basis of the aforementioned evidences, HNK is a promising compound to be exploited to attenuate cisplatin-induced renal toxicity and to improve clinical safety of cisplatin for patients who undergo cancer treatments. In this study, we aim to evaluate *in vitro*, the short-term (within 24 h treatment) effects of HNK on cisplatin-induced damages using renal epithelial cells as a testing model system.

## Materials and Methods

### Chemicals, Reagents, Antibodies

Chemicals and reagents were obtained from Sigma-Aldrich (St. Louis, MO, United States) unless otherwise stated. *Cis*-Diammineplatinum(II) dichloride (Cisplatin, Cat. #479306, purity ≥ 99.9%) was purchased from Sigma. 2-(4-hydroxy-3-prop-2-enyl-phenyl)-4-prop-2-enyl-phenol (Honokiol, Cat. #SLK S2310, purity: 99.81%) was obtained from Selleckchem (Houston, TX, United States). Mouse monoclonal anti-E-Cadherin antibody (for immunofluorescence staining) was obtained from Cell Signaling Technology Inc. (MA, United States), rat monoclonal anti-E-Cadherin antibody (for Western-Blotting) was obtained from Sigma. Rabbit polyclonal anti-occludin antibody was purchased from Santa Cruz Biotechnology (Santa Cruz, CA, United States), mouse monoclonal anti-8-OHdG antibody and anti-GAPDH antibody was acquired from Abcam (Cambridge, United Kingdom) and Ambion (Invitrogen/Life Technologies, Carlsbad, CA, United States), respectively. For cytoskeleton detection and quantification, Alexa 568-Phalloidin and Alexa 488-anti-α tubulin antibodies were used (Invitrogen/Life Technologies). All secondary antibodies were purchased from Jackson ImmunoResearch (Jackson ImmunoResearch Laboratories Inc., West Grove, PA, United States).

### Cell Culture

Stable cell line of Madin Darby Canine Kidney epithelial cell (MDCK) was purchased from American Type Culture Collection (ATCC, PTA-6500, Manassas, VA, United States) used throughout the study. Cells were cultured in Dulbecco’s Modification of Eagle’s Medium (DMEM, Gibco, NY, United States) supplemented with 10% fetal bovine serum (FBS) and 1% penicillin-streptomycin-amphotericin B (Gibco) at 37.5°C in humidified atmosphere with 5% CO_2_ for at least five passages before use for experiments. For whole cell lysate, after treatments, cells were rinsed with ice-cold phosphate-buffered saline (PBS) (Gibco) and subsequently scraped into RIPA lysis buffer (Boston Bio Products, Ashland, MA, United States) supplemented with protease inhibitors (EDTA free, Roche, Mannheim, Germany). Cells were lysed on ice for 1 h and were passed through a 25-gauge syringe followed by 30 second sonication in ice-cold water bath. Protein quantification was carried out with Pierce^®^ BCA Protein Assay Kit (Pierce Biotechnology, Rockford, IL, United States), lysed cells were flash-frozen in liquid nitrogen and stored at -80°C until use.

### Cell Viability Assay (MTT Assay)

To determine cell toxicity of cisplatin and HNK, cell viability assay namely (3-(4,5-Dimethylthiazol-2-yl)-2,5-Diphenyltetrazolium Bromide) (MTT), (Sigma-Aldrich) assay was carried out. In brief, MTT stock (5 mg/ml in PBS) was prepared and filtered through 0.22 μm polyvinylidene fluoride (PVDF) membrane filter (Millipore) and was subsequently stored at -20°C for maximum 1 week. To perform MTT assay, MDCK cells were seeded in sterile 96-well plate in the density of 750 cells/well and were grown for 3 days. Cells were serum-starved for 2 h prior to the treatments. Four hours before the end of treatments, 10 μl MTT was added into each well (final concentration of 0.5 mg/ml, 1.2 mM) and incubated at 37.5°C. Unbound MTT was removed from the medium and cell-bound MTT was extracted with 100 μl dimethyl sulfoxide (DMSO) for 1.5 h at room temperature (RT). After the cell-bound MTT crystals were resolved, optical density (OD) was measured at 570 nm, and background values were measured using 650 nm with a microplate reader (SpectraMax M5, Molecular Devices, United States).

### Immunoblotting

To evaluate the effects of cisplatin and HNK on the protein expression level of Occludin and E-cadherin, immune-blotting experiments were performed as preciously described (Kuo et al., 2017). Proteins were separated by SDS-PAGE and wet-blotted onto an Immobilon-P PVDF membrane (Millipore, MA, United States). Non-specific signals were minimized with blocking buffer (5 mM Tris, 250 mM sucrose, pH 7.4 with 0.05% v/v Tween-20 [TBST], supplemented with 5% milk powder). Primary antibody (E-Cadherin 1:250, Occludin 1:500, GADPH 1:10000) and secondary antibody (1:5000) were subsequently used as previously described (Kuo et al., 2017). Protein signals were visualized by chemiluminescence (Merck Ltd., TW) and were detected with ChemiDoc^TM^ XRS+ system (Bio-Rad).

### Indirect Immunofluorescence Staining and Image Acquisition

For indirect immunofluorescent staining, cultured cells were rinsed and fixed with 4% (v/v) paraformaldehyde (PFA) at RT for 30 min. For Occludin staining, additional pre-extraction procedure was required. Cells were incubated with pre-extraction buffer [0.2% Triton X-100, 100 mM KCl, 3 mM MgCl_2_, 1 mM CaCl_2_, 200 mM sucrose, 100 mM Hepes (pH 7.1)] for 2 min at 4°C prior to fixation. Fixed cells were washed and permeabilized with 0.1% (v/v) Triton X-100 for 4 min on ice and subsequently blocked in blocking buffer (PBS supplemented with 1% (w/v) BSA) for 30 min at RT. Antibody incubations were carried out as described above (E-Cadherin 1:1000, Occludin 1:1000). Stained cells were mounted with Vectashield in the presence of diamidino-2-phenylindole (DAPI, Vector Lab, Peterborough, United Kingdom). All samples were evaluated with either Olympus IX83 epifluorescent microscopy or with Leica TCS SP5 II confocal scanning microscopy. Background subtraction and contrast/brightness enhancement (up to ∼20% enhancement using the maximum slider in both software) were performed identically for all images in the same experiment. For *in vitro* OS evaluation, after required treatments, cells were fixed and stained with anti-8-OHdG antibody (1:1000). Positive cells were manually counted and the percentages of positive cells were calculated accordingly. For signal correlation analysis between Occludin and E-cadherin, 10 images were randomly taken from each group, Pearson’s correlation coefficients were calculated using ImageJ (NIH^1^).

### 2D Polarized Transwell^®^ Culture and Signal Dispersion Analysis

Madin Darby Canine Kidney cells were seeded in the Transwell^®^ (pore size 0.4 μm) at the density of 80,000 cells/well and were grown for overnight to achieve 100% confluence of monolayer. The cells were serum-starved and treatments were added in both the upper and the lower chamber. Immunofluorescent staining on polarized cells was carried out as in regular cultured cells described above. After staining, the Transwell^®^ membranes were excised and placed on glass slides for microscope evaluation. To generate 3D information on monolayer images from 2D Transwell^®^ culture, polarized cells were scanned with Leica TCS SP5 II confocal scanning microscopy using 100X object with a step size of 0.3 μm. For junction protein dispersion analysis, the z-stack images were re-sliced along the *X*-axis and the re-sliced images were superimposed using Z-project function in ImageJ to generate lateral views of each single cell layer for further quantification.

### Cell Spreading Assay

The MDCK cells were cultured and prepared as above-mentioned except the growth surface was coated with fibronectin (0.1 μg/ml, Sigma) to enhance cell attachment. Cells were serum-starved and seeded into a 12-well plate in the presence of either 10 μM of cisplatin, 10 μM of HNK or 10 nM latrunculin B. Cells were allowed to spread at 37.5°C for required time prior to 4% PFA fixation. After fixation and permeabilization, cells were labeled with Alexa-568 Phalloidin and DAPI to observe polymerized F-actin structure and cell nucleus. Cell spreading ability was quantitatively accessed by a self-defined “cell-spreading index.” Cell spreading index was defined as the ratio between total cell surface and cell nucleus surface. Cell spreading index was then accessed by manual selection of ∼100 cells/group, total cell membrane surface and cell nucleus surface were measured and calculated using ImageJ.

### Cytoskeleton Quantification and Characterization

To quantify the amount of polymerized microfilament, F-actin assay was performed as previously described (Yui et al., 2013). Single cell suspension was added onto 24-well plate, and cells were allowed to grow in complete DMEM (10% FBS, 1% penicillin-streptomycin) for 3 days. Cells were serum-starved prior to treatments, and after the treatments, cells were rinsed with PBS and incubated with Hepes binding buffer (20 mM KH_2_PO_4_, 10 mM HEPES, 5 mM EGTA, 2 mM MgCl_2_, 0.1% Triton X-100, pH 7.4), containing Alexa 568- Phalloidin (250 nM) and 4% PFA, for 20 min at RT. Negative controls were carried out by incubations in the absence of Phalloidin. Latrunculin B treatment was used as positive control to reach the maximal effect for depolymerization of F-actin. To extract F-actin bound Alexa 568-Phalloidin, cells were subsequently incubated with pure methanol (300 μl/well) for overnight at -20°C. The extracted Alexa 568-Phalloidin fluorescence was transferred to opaque 96-well plates and read on a SpectraMax M5 multiplate reader (excitation 568 nm, emission 618 nm, cutoff 610 nm).

Quantification of polymerized α-tubulin was followed by method described by Sum et al. (2014). In brief, MDCK cells were seeded on the coverslips and were grown for 2–3 days to achieve an 80% confluence. Cells were serum-starved and treated with 10μM cisplatin, 10 μM HNK or 10 μM colchicine. After the treatments, the tubulin bundle was labeled with anti-α-tubulin antibody conjugated with Alexa 488 and imaged with Olympus IX83 microscopy. At least 10 pictures were randomly taken from each group. All images were analyzed with the CellSens software (Olympus, Tokyo, Japan). Area exceeding a designed threshold (same for all conditions) was quantified and divided by the total cell surface area to represent the quantity of polymerized tubulin bundle within a cell. The data were averaged in each group and were presented as relative fold changes compared to vehicle control groups of each experimental condition.

### Cellular Anti-oxidation Ability and Mitochondria Status Assessment

To evaluate HNK and cisplatin effects on cellular redox status and mitochondria function, commercially available OxiSelect^TM^ assay kit for total antioxidant capacity (TAC) measurement (Cell Biolabs, Inc., San Diego, CA, United States) and MitoTracker^®^ Red CM-H_2_XRos dye for mitochondria oxidation status were used. For TAC assay, standard manufactory instructions were followed. In brief, total cell lysates from each treatment were added into a 96-well microtiter plate and mixed with reaction buffer. For kinetic measurement, after 4 h treatments, cells were lyzed and processed as described above, 96-well microtiter plate was placed immediately into the SpectraMax M5 multiplate reader and optical density (OD) values were obtained for consecutive 30 min with 1 min interval. Endpoint TAC measurement was carried out using cells treated for 24 h. To initiate the reaction, Copper ion reagent was added into each well and incubated for required time on shaker. Stop solution was subsequently added into the well before reading of OD values at 490 nm. For mitochondria status evaluation, cells were grown, starved and treated as above described. After treatments, cells were rinsed with serum free DMEM before the incubation of 500 nM MitoTracker^®^ Red CM-H_2_XRos for 30 min at 37.5°C. After staining, cells were rinsed and fixed subsequently with 4% PFA and cell nuclei were stained with DAPI. One hundred cells were manually counted and mitochondria health was evaluated as the % of cells with strong intensity for MitoTracker^®^ Red CM-XRos as only cells with diminished mitochondria oxidation status exhibited strong fluorescent signal for MitoTracker^®^ Red CM-XRos.

### Statistical Analyses

Results were expressed as mean ± standard error of mean (SEM). Comparative studies of means were performed using a one-way analysis of variance (ANOVA) followed by a Kruskal–Wallis test. Significance was set with *p* < 0.05.

## Results

### Honokiol Attenuated Cisplatin-Induced Disorganization of Occludin and E-Cadherin

**FIGURE 1:**
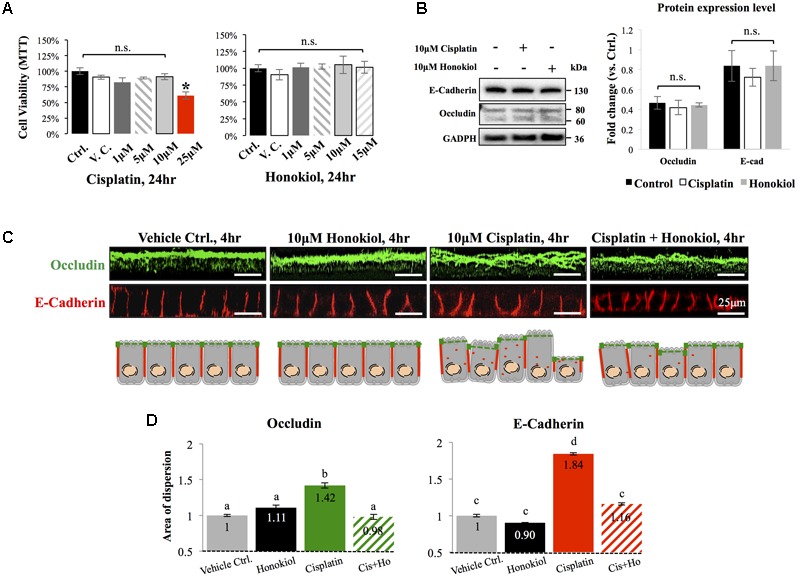
Cytotoxicity analyses and the effects of cisplatin and honokiol (HNK) on protein expression and cellular localization of Occludin and E-Cadherin. **(A)** To determine cell toxicity of cisplatin and HNK, cell viability assay namely MTT [(3-(4,5-Dimethylthiazol-2-yl)-2,5-Diphenyltetrazolium Bromide)] assay was carried out. Up to 10 μM of cisplatin and 15 μM of HNK, no cytotoxicity was detected. **(B)** Western-blotting was performed to evaluate the effect of HNK on the protein expression of E-Cadherin and Occludin. **(C)** Two-dimension polarized cell culture system showed cisplatin treatment induced redistribution of tight junction protein Occludin (in green) from the apical membrane region toward the cytosol as disorganized and multiple layers of signals were observed. Similar to that of Occludin, E-Cadherin (in red) moved from the lateral membrane toward the cytosol as a thicker E-Cadherin signal and increased cytosolic E-Cadherin signal were observed upon cisplatin treatment. This redistribution of junction proteins was partly decreased in the HNK co-incubation group. Cartoon images depicted and summarized the observed phenomenon. **(D)** Signal dispersion analysis showed in cisplatin-treated group, both Occludin and E-Cadherin are more dispersed into the cytosol than control or HNK-treated cells (1.42- and 1.84-fold more dispersed for Occludin and E-Cadherin, respectively). At least 3–5 independent experiments were performed. N.S. indicated no statistical difference. Asterisk indicated significant difference at *p* < 0.05. a–d, indicated significant (*p* < 0.05) difference between groups. Data were expressed as fold change as compared with control condition.

**FIGURE 2:**
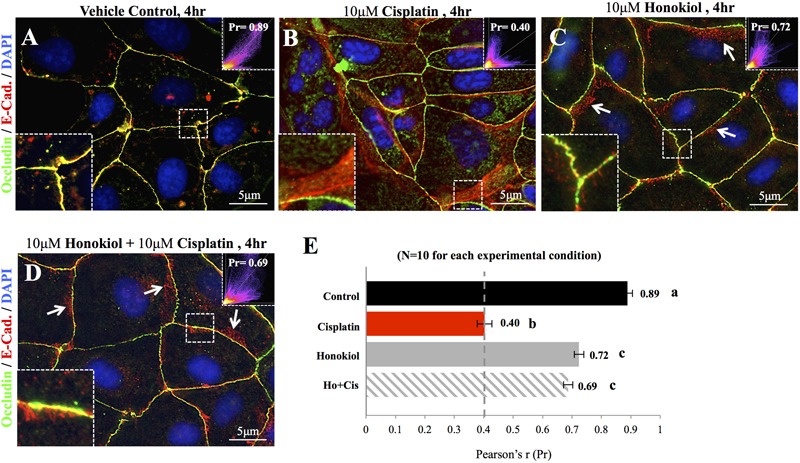
Co-localization analyses of E-Cadherin and Occludin. Co-localization of E-Cadherin (in red) and Occludin (in green) was analyzed on flat cell culture. Co-localization of two proteins can be visualized in yellow and quantified with Pearson’s correlation coefficient (Pr) from 10 randomly took images in each group **(E)**. **(A)** Control cells showed high degree of co-localization with Pr value of 0.89, **(B)** When cells were treated with 10 μM cisplatin, both Occludin (in green) and E-Cadherin (in red) can be detected in the cytosol. Enlarged imaged showed the movement of E-Cadherin from the lateral membrane into the cytosol. Pr value decreased from 0.89 in control cells to 0.4 in cisplatin-treated cells indicated the decreased in co-localization of two signals. **(C,D)** When HNK alone **(C)** or was co-incubated with cisplatin **(D)**, Pr value increased from 0.4 to 0.72 and 0.69, respectively, indicated an increased co-localization of E-Cadherin and Occludin. Enlarged magnification showed a minor degree of E-Cadherin (in red) moved away from the lateral membrane and these redistributed signals exhibited in punctate vesicle-like signals (indicated with arrows). Images presented were representative images, and at least 10 images from each group were taken randomly and assessed. a–c indicated significant (*p* < 0.05) difference between groups.

To investigate the effects of HNK, we first examined HNK effect on protein expression and cellular localization of E-Cadherin and Occludin, two proteins located at the adhesion and tight junction of kidney epithelial cells, respectively. As showed in **Figure 1A**, up to 10 μM of cisplatin and 15 μM of HNK, no cytotoxicity was detected, we thereafter applied 10 μM concentrations for both compounds in all subsequent experiments. Moreover, we observed no changes in cell viability under 10 μM HNK/10 μM cisplatin combination in 24 h- MTT assay (data not showed) indicated no apparent cytotoxicity from this combined incubation. We detected no significant changes on protein expression level for both E-Cadherin and Occludin upon cisplatin or HNK treatments (**Figure 1B**); however, when cells were grown in polarized 2D Transwell^®^ system, an apparent redistribution of both proteins was noted (**Figure 1C**). Occludin (in green) and E-Cadherin (in red) redistributed from the apical or lateral side of MDCK cells toward the cytosol upon cisplatin treatment; however, when HNK was co-present with cisplatin, the disorganized signals of both proteins were partially inhibited (**Figure 1C**). Quantification analyses further confirmed our observation that when compared with control or HNK-treated cells, Occludin and E-Cadherin were 1.42- and 1.84- fold more dispersed into the cytosol in those of cisplatin-treated cells. Moreover, HNK co-incubation with cisplatin significantly reduced the dispersion of both proteins (**Figure 1D**, stripped bars). In agreement with our observations, co-localization analysis on epi-fluorescent images indicated that cisplatin treatment reduced the co-localization of Occludin and E-Cadherin signals (**Figures 2A–C**); however, when HNK was present in the cisplatin-containing medium, co-localization of two proteins and Pearson’s correlation can partly be restored (**Figure 2D**). Pearson’s correlation analyses showed a restoration value from 0.4 to 0.69 with a higher degree of co-localized signal (in yellow) when HNK was co-present in the cisplatin-containing incubation medium (**Figures 2D,E**). The reduced co-localization of two junction proteins and the increased cytosol detection of both Occludin and E-Cadherin suggested the internalization of both proteins.

### Honokiol Promoted the Polymerization of Actin Cytoskeleton

**FIGURE 3:**
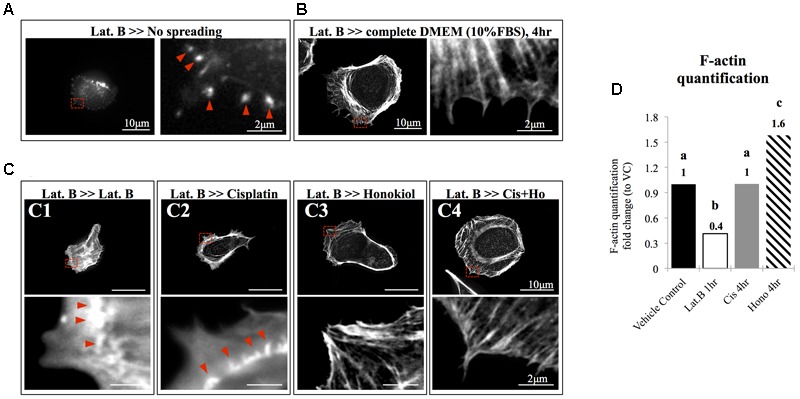
The effects of honokiol on actin polymerization. To evaluate the effects of cisplatin and HNK on the polymerization of actin cytoskeleton, maximum de-polymerization was achieved by incubation of cells in 10 nM of latrunculin B for 4 h. **(A)** When cells were not allowed to recover, F-actin aggregates (marked with red arrowheads) were observed at the edge of the cells and no elongation of F-actin was observed. **(B)** When cells were allowed to recover in a complete culture medium supplemented with 10% FBS, a complex actin network can be observed at the leading edge of the cells and stress fibers were also observed. Enlarged image showed elongated actin fiber extended toward the cell edge. **(C)** After cells were de-polymerized and were allowed to recover (for overnight) in culture medium containing, **(C1)** 10 nM of latrunculin B and **(C2)** 10 μM of cisplatin, minimal amount of elongated actin was detected. Enlarged image showed that unlike in full recovery medium, short polymerized actin fiber or aggregates were observed at the cell edge (marked with red arrowheads). However, when HNK was present alone **(C3)** or was co-present in the recovery medium **(C4)**, the complex actin network and the elongated actin cytoskeleton or stress fiber were observed at the cell edge. **(D)** F-actin assay demonstrated cisplatin did not caused de-polymerization of actin; and HNK promoted (increased 1.6-fold) the polymerization of actin cytoskeleton. Images presented were representative images, and at least 20 images from each group were taken randomly and assessed. a–c indicated significant (*p* < 0.05) difference between groups. Lat. B, latrunculin B.

**FIGURE 4:**
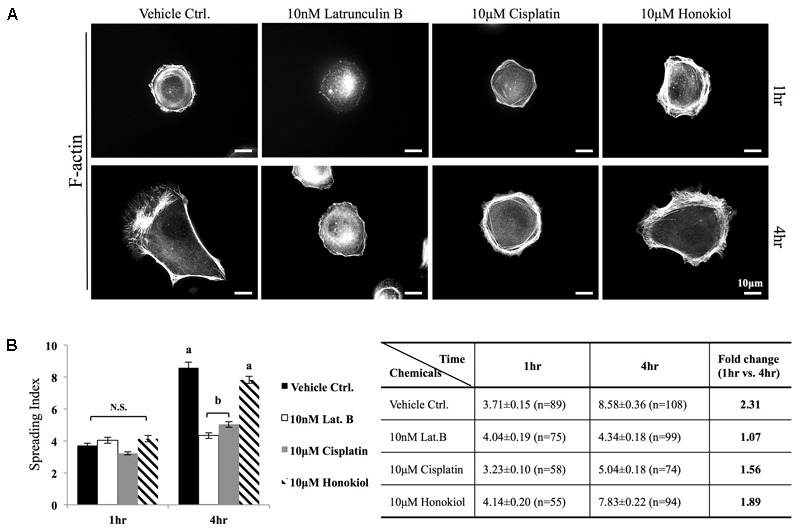
Cell spreading assay. **(A)** MDCK cells were trypsinized, re-seeded on a fibronectin (0.1 μg/ml, Sigma) coated coverslips were allowed to spread at 37.5°C for required time (1–4 h) in the medium containing either 10 μM of cisplatin, 10 μM of HNK or 10 nM latrunculin B. F-actin was labeled with Phalloidin-Alexa 594. **(B)** Cell spreading ability was quantitatively accessed by a self-defined “cell-spreading index”. Cell spreading index was then defined as the ratio between total cell surface and cell nucleus surface and around 100 cells/group were measured. Within a 4 h cell spreading time, control cells spread and expanded 2.31-fold when compared with 1 h. When cells were spreading in the presence of latrunculin B (Lat. B), no expansion of cell surface was measured. When cells were allowed to spread in the presence of cisplatin, a 1.5-fold increase in cell surface area was measured; however, this ratio was not significantly differed from negative control (Lat. B) group. A significant increase of cell surface (1.89-fold) was measured the 10 μM of HNK was present in the medium. Images presented were representative images. a,b indicated significant (*p* < 0.05) difference between groups. NS, non-statistically different.

The morphology of the epithelial cells relies on structural organization of polymerized cytoskeleton, we showed in **Figure 3A** that when cells treated with latrunculin B for 24 h, a compound that prevents the polymerization of actin, a minimal amount of F-actin was detected; moreover, remnants of actin aggregates were observed at the edges of the cells (indicated with red arrowheads); however, when cells were allowed to recover in a complete DMEM (supplied with 10% FBS) for 4 h after maximum de-polymerization with latrunculin B, the complexity of polymerized actin network and the stress fibers were observed (**Figure 3B**). In contrast, after maximum de-polymerization, when recovery medium contained 10 μM latrunculin B or cisplatin, short actin aggregates were observed (**Figures 3C1,C2**); however, when HNK is (co)-present in the recovery medium, a complex actin network as well as stress fibers can again be observed (**Figures 3C3,C4**). F-actin quantification analysis showed no significant reduction on the amount of F-actin upon cisplatin treatment; however, when HNK is present, a 1.6-fold increase in polymerized F-actin was detected (**Figure 3D**). This HNK effects on polymerized actin might explain our observation on cell spreading ability that HNK-treated cells showed similar cell spreading ability as control cells while cisplatin-treated cells exhibited a reduced spreading ability (**Figure 4**). From cell spreading ability assay, after 4 h spreading time, HNK-treated cells could extend 1.89-fold on its surface area, showed slightly less, but not statistical different from those of control cells (2.31-fold); however, in cisplatin-treated cells, only 1.56-fold on cell surface extension was measurement (**Figure 4B**). These data demonstrated that in contrast to HNK promotes the polymerization of actin, cisplatin, although did not lead to de-polymerization of actin cytoskeleton, but did retard the polymerization process of G actin into functional polymerized F-actin.

### Honokiol Promoted the Polymerization of Tubulin and Increased the Formation of Tubulin Bundle

**FIGURE 5:**
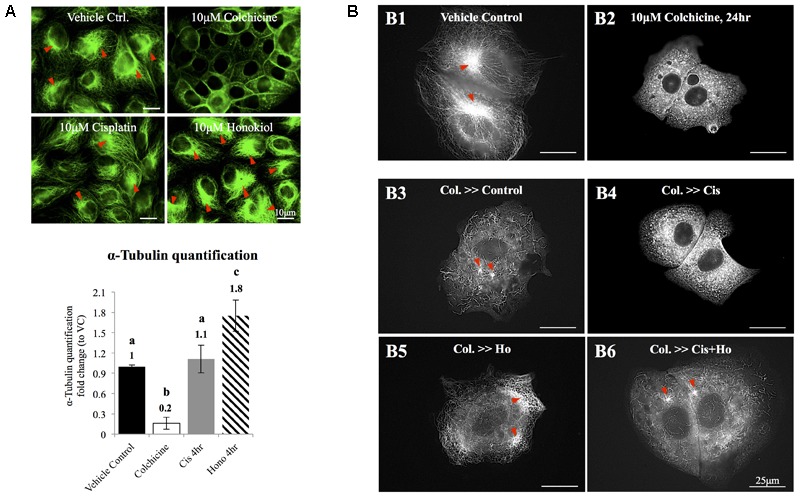
The effects of honokiol on tubulin. **(A)** To evaluate the effects of cisplatin and HNK on the polymerization of tubulin, maximum de-polymerization was achieved by incubation of cells in 10 μM of colchicine for 24 h. Tubulin bundles were visualized and marked with red arrowheads. When cells were treated with cisplatin, no change on tubulin bundle was observed; however, when cells were treated with HNK, significant 1.8-fold increase of tubulin bundle was measured. **(B,B1)** Control cells showed complexity of tubulin network with apparent microtubule organization centers (marked in red arrowheads). **(B2)** Cells recovered in medium containing 10 μM colchicine showed punctate tubulin signal in the cytosol, and **(B3)** when cells were allowed to recover (for 4 h) in culture medium containing 10% FBS (complete culture medium), tubulin bundle as well as microtubule organizing center can be observed again. **(B4)** When cells recovered in the presence of 10 μM cisplatin, punctate tubulin signals were observed. However, when HNK was present either alone **(B5)** or was co-present in the recovery medium **(B6)**, elongated tubulin and microtubule organizing center (marked with red arrowheads) can be observed. Images presented were representative images, and at least 20 images from each group were taken randomly and assessed. a–c indicated significant (*p* < 0.05) difference between groups. Lat. B, latrunculin B.

Redistribution of cellular components relies on organized microtubules; we next examined the effects of HNK on microtubule. As showed in **Figure 5A**, cisplatin did not cause de-polymerization of tubulin; however, when HNK was present, a 1.8-fold increase in total amount of α-tubulin bundle was detected (**Figure 5A**, tubulin bundles were marked with red arrowheads). When cytoskeleton recovery experiments were performed, cells were pre-treated with, a compound that inhibits the polymerization of tubulin. When compared with control cells (**Figure 5B1**), we showed that 10 μM colchicine-treated cells (for 24 h) exhibited punctate tubulin signals in the cytosol indicated the depolymerized tubulin fragments (**Figure 5B2**), and when cells were allowed to recover in the complete recovery medium (DMEM with 10% FBS) for 4 h, reappearance of polymerized tubulin and microtubule organizing center were observed (**Figure 5B3**, marked with red arrowheads). When cells were recovered in the presence of 10 μM cisplatin for 4 h, no elongated tubulin was observed (**Figure 5B4**); in contrast, in both HNK alone or cisplatin/HNK co-incubation group, an increased amount of elongated tubulin with microtubule organizing centers were observed (**Figures 5B5,B6**, marked with red arrowheads), suggested HNK promoted the polymerization process of tubulin in MDCK cells.

### Honokiol Reduced Cisplatin-Induced Oxidative Stress

**FIGURE 6:**
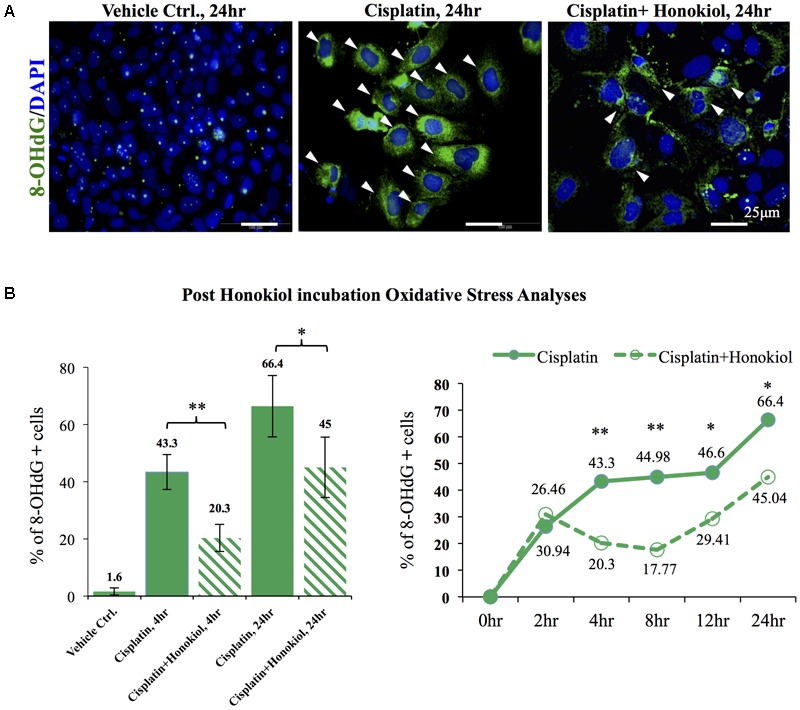
*In vitro* and *in vivo* oxidative stress assessments. **(A)** Oxidative stress marker protein 8-OHdG was used to detect cisplatin-induced damages (positive cells were marked in green with arrowheads). Cisplatin treatment increased detection of 8-OHdG while co-incubation of HNKl significantly reduced cellular oxidative. **(B)** Quantification analysis showed that cells that were positive for oxidative stress decreased from 44.3 to 20.3% and from 66.4% in cisplatin-treated group to 45% when HNK was co-incubated. Time-dependent anti-oxidant analyses revealed significant reduction of 8-OHdG positive cells in HNK-treated group, and the peak effect appeared at 8 h after the incubation and gradually diminished after 12–24 h. Data were presented as mean ± SEM. Images presented were representative images. Statistical significant difference between groups were marked with ^∗∗^*p* < 0.01, ^∗^0.05 < *p* < 0.1.

As showed in **Figure 6**, a minimal amount of OS marker protein 8-Hydroxydeoxyguanosine (8-OHdG) was observed in control groups while an increased 8-OHdG signal was detected in cisplatin-treated cells (**Figure 6A**); however, this increase of 8-OHdG was abated when these cells were co-incubated with HNK (**Figure 6A**). Furthermore, a time-dependent increase in cisplatin-induced OS was observed, and this OS was significantly reduced (from 43.3 to 20.3% at 4 h and 66.4 to 45% at 24 h) upon HNK co-incubation (**Figure 6B**). Time-dependent analyses revealed that anti-oxidant effect of HNK started from 4 h after the co-incubation and peak effect at 8 h (27.21% reduction of 8-OHdG^+^ cells when compared with cisplatin alone group), and this protective effect gradually diminishing after 12–24 h (**Figure 6B**). These data demonstrated for the first time that HNK could reduce cisplatin-induced OS in renal epithelial cells.

### Honokiol Maintained Cellular Antioxidant Ability and Maintained Cellular Oxidation Ability

**FIGURE 7:**
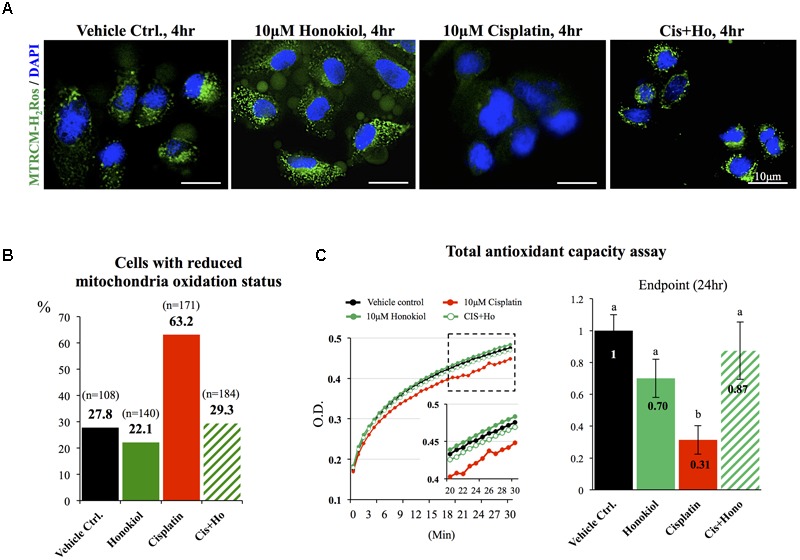
Mitochondria status and total anti-oxidant ability assessments. **(A)** Specific formulation of MitoTracker^®^ Red CM-H_2_XRos was used to identify cells with diminished mitochondria oxidation ability. Control MDCK cells showed weak signal similar to those of HNK-treated and cisplatin-HNK co-incubation cells while cisplatin-treated cells exhibited strong fluorescent signal indicating diminished oxidation status of mitochondria in these cells. **(B)** Quantification of cells with diminished oxidation status demonstrated significant increase of cells with diminished oxidation status in cisplatin-treated group, and this negative effect can be attenuated when 10 μM HNK was co-incubated in the presence of 10 μM cisplatin. **(C)** Total anti-oxidant capability (TAC) assay was performed to access cellular anti-oxidant ability. Kinetic TAC measurement at the first 30 min after the initiation of the assay showed a clear reduction of total antioxidant capacity in cisplatin-treated cells (in red). Based on endpoint measurement, when compared with vehicle control, cisplatin-treated cells showed sharp reduction on TAC (69% reduction), and this reduction was attenuated when Honokiol was con-present in the incubation medium. Data were presented as mean ± SEM, and representative images were presented. a,b indicated significant (*p* < 0.05) difference between groups.

It is known that cisplatin induces OS by generating excessive amounts of ROS and disturbs mitochondria antioxidant ability (Townsend et al., 2009; Miller et al., 2010). When we applied mitochondria dye MitoTracker^®^ Red CM-H_2_XRos to examine the oxidation status of mitochondria, we observed a sharp increase in the number of MitoTracker^®^ Red CM-H_2_XRos negative cells in cisplatin-treated (63.2%) group when compared with either control (27.8%) or HNK-treated cells (22.1%). And this reduction of cellular oxidation ability was attenuated by HNK co-incubation with cisplatin (**Figures 7A,B**, cells with diminished mitochondria oxidation ability decreased from 63.2% in cisplatin group to 29.3% in co-incubation group). Furthermore, kinetic measurement on total antioxidant capability demonstrated a clear reduction on total antioxidant capability in cisplatin-treated group (in red) when compared with control (in black), honokiol alone (in dark green) or cisplatin-honokiol co-incubation (in light green) group (**Figure 7C**). Our end point total antioxidant capacity assay (at 24 h treatment) revealed that cisplatin diminished the total antioxidant capacity in MDCK cells (reduced 69%, **Figure 7C**, right panel) and HNK could reversed cisplatin-induced reduction of antioxidant capacity to a level comparable to those of control cells (**Figure 7C**).

## Discussion

In this study, we showed that HNK attenuated cisplatin-induced oxidative stress in kidney epithelial cells, and moreover, HNK was able to reduce cisplatin-induced disorganization of E-Cadherin and Occludin. Stabilization of these junction proteins at their original cellular locations was likely resulted from HNK effect on promoting the polymerization of actin and tubulin that are connected with these junction protein complexes.

Cisplatin forms DNA adducts, and once enters the cells, the subsequent DNA damages lead to cell cycle arrest and cell apoptosis (Desoize and Madoulet, 2002; Chaney et al., 2005; Garcia Sar et al., 2012; Karasawa and Steyger, 2015). Despite its anti-cancer ability, nephrotoxicity is one of the main dose-limiting side effects that often prevent systemic cisplatin administration at its full efficacious dose (Litterst et al., 1976; Kuhlmann et al., 1997). Numbers of cisplatin analogs have been synthesized to reduce organ-toxicity; however, these modified analogs exhibit less antineoplastic efficacy with other unwanted sides effects (Dasari and Tchounwou, 2014). Cisplatin-induced renal toxicity resulted from the accumulation of cisplatin in renal tubules, as cisplatin concentration is about five times higher in renal tubules than in serum (Vickers et al., 2004). This accumulation was likely due to the imbalance between cisplatin intake (by organic cation transporter 2) and output (by multidrug and toxin extrusion proteins) transporters at the renal tubule epithelium (Vickers et al., 2004; Wagner et al., 2016). From *in vitro* studies, cisplatin has been shown to provoke mitochondrial dysfunction by interfering the electron transfer system and thus enhances the generation of ROS and RNS (Choi et al., 2015). Moreover, cisplatin binds to and inhibits the activities of glutathione or other antioxidant enzymes including glutathione peroxidase (GPX) and superoxide dismutase (Yasuyuki et al., 1992; Khynriam and Prasad, 2002). By enhancing ROS production with decreasing antioxidant enzyme activities, cisplatin disrupts the cellular redox balance and induces OS. It has been shown that HNK effectively inhibits both the initiation and the propagation of lipid peroxidation and thus possess anti-oxidation property via the ability to reduce ROS production (Dikalov et al., 2008; Yu et al., 2016). *In vitro* experiments demonstrated that HNK inhibited xanthine oxidase, suppressed NADPH oxidase activity, and increased GPX activity (Liou et al., 2003). Moreover, HNK itself is also an effective free radical scavenger that can efficiently scavenge superoxide radicals (Dikalov et al., 2008). In this study, we demonstrated that HNK reduced cisplatin-induced OS *in vitro*, the reduction of OS in the renal epithelial cells was resulted from the maintenance of mitochondria oxidation ability and total cellular anti-oxidant capacity, although HNK may not reduce the amount of ROS and RNS generated by cisplatin; however, maintaining mitochondria antioxidant enzyme activity and its oxidation ability would attenuate cellular redox imbalance caused by cisplatin which consequently resulted in the improvement of renal epithelial cell health and functions. It is worth mention that we observed a gradual decrease in HNK protection against cisplatin-induced oxidative stress after 12–24 h, this are likely due to the fast half-life of HNK as mentioned in other studies (40–60 min for intravenous injection and 4–6 h for intraperitoneal injection) (Tsai et al., 1994). As no current data is available on the serum or renal tubular concentrations of HNK from *in vivo* experiments, therefore, whether HNK could reach an effective serum concentration in the target organ and provides sufficient protective effect *in vivo* require further investigation and pharmacokinetics analysis.

**FIGURE 8:**
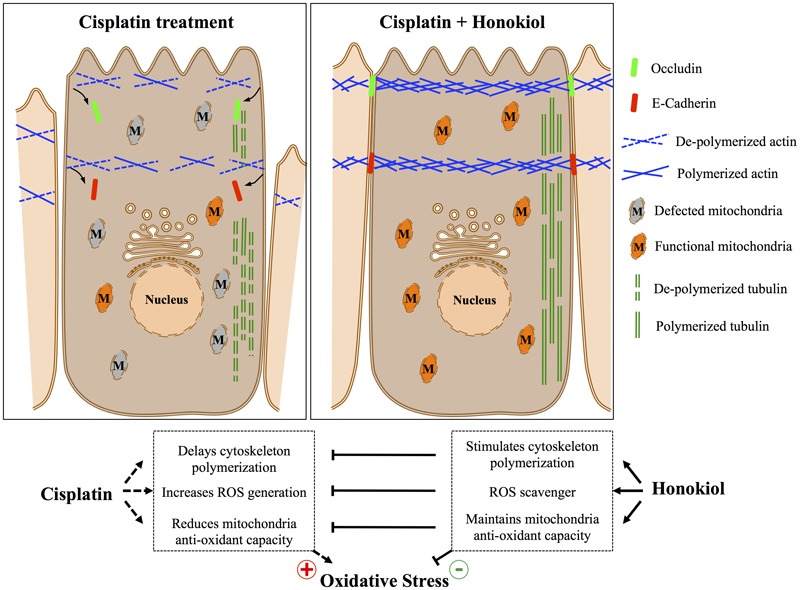
Schematic summary of Honokiol attenuation on cisplatin-induced cellular damages.

It is known that junction proteins (e.g., tight junction protein Occludin, claudins or adhesion junction protein E-Cadherin and β-catenin) together with polymerized cytoskeletal network are critical to maintain epithelial cell polarity, disruption or disorganization of junction protein complexes from their cellular localization results in loss of epithelial cell polarity and leads to kidney tubule dysfunction (Fish and Molitoris, 1994; Giepmans and van Ijzendoorn, 2009; Mullins and Hansen, 2013). In contrast to earlier report by Trujillo et al. (2014) that cisplatin reduced protein expression of tight junction proteins Occludin and claudin 2, we observed no reductions on the protein expression level for either E-Cadherin or Occludin after cisplatin treatment; however, we observed dis-localization of both proteins induced by cisplatin, and HNK treatment partially reduced this redistribution phenomenon. Stabilization of junction proteins rely on polymerized actin cytoskeleton connected to junction protein complexes (Jefferson et al., 2004), disruption of cytoskeletal network results in disorganized cellular proteins and disruption of cell polarity. To our surprise, cisplatin did not cause actin nor tubulin de-polymerization under our experimental setups, instead, from our series cytoskeleton recovery experiments, we observed that cisplatin retarded the polymerization process of both cytoskeleton after maximum de-polymerization of microfilament and microtubule. Short actin aggregates and punctate microtubule signals were observed in cisplatin-treated cells and this may further explain the defected or delayed cell spreading ability in these cells. In a sharp contrast, when cells were allowed to recover in the presence of HNK, renal epithelial cells exhibited a complex cytoskeletal network with elongated microtubules within the same period of recovery time demonstrated that HNK not only promoted the polymerization of cytoskeleton, but also counteracted with the effects of cisplatin. This HNK effects on the polymerization of cytoskeleton were likely accounted for the maintenance of E-Cadherin and Occludin at their cellular localization. Although in this particular study, we are not able to distinguish whether the maintenance of cytoskeleton was a consequence resulted from the reduced oxidative damage; however, this is, to our knowledge, the first report demonstrated that HNK promoted the polymerization of cytoskeleton and maintained cellular localization of junction proteins that are responsible for cell polarity. A schematic summary of Honokiol effects on renal epithelial cells is illustrated in **Figure 8**.

It is known that the cytotoxicity effect of cisplatin is more apparent in the fast dividing and proliferating cells. Currently, no direct evidence indicates the counteracting effect of HNK on the anti-cancer capacity of cisplatin, in contrast, published literatures demonstrated that the combination of HNK with cisplatin exhibited synergic effect on cancer treatments (Jiang et al., 2008; Cheng et al., 2011). From our cytotoxicity evaluations on HNK and cisplatin co-incubation, we observed no significant cytotoxicity when 10–25 μM HNK was combined with 10 μM cisplatin. However, when HNK concentration was higher than 25 μM, changes in cell morphology and cell viability were observed.

## Conclusion

We demonstrated for the first time, the protective effects of HNK against cisplatin-induced cellular damages in kidney epithelial cells, and these positive outcomes were likely resulted from the combined effects of the (1) decreased ROS production and thus maintain cellular redox balance and cellular oxidation ability, and (2) stabilization of junction proteins via the modulation of and (3) the maintenance of cytoskeletal structure. Future *in vivo* study will further validate the potential clinical applications of HNK in cisplatin-based cancer treatments with reduced cellular toxicity.

## Author Contributions

T-EW, H-TL, and Y-HL carried out all the experiments presented in this manuscript. T-RJ, NN, C-CC, and Y-JL conceived and designed the experiments. T-EW, Y-HL, and H-WC analyzed the data. H-WC and P-ST wrote and constructed the manuscript. All authors read and approved this article at submission.

## Conflict of Interest Statement

The authors declare that the research was conducted in the absence of any commercial or financial relationships that could be construed as a potential conflict of interest. The reviewer AD and handling Editor declared their shared affiliation.

## Footnotes

1 http://rsb.info.nih.gov/ij/
